# Multidimensional frailty and quality of life: data from the English Longitudinal Study of Ageing

**DOI:** 10.1007/s11136-022-03152-9

**Published:** 2022-05-17

**Authors:** Nicola Veronese, Marianna Noale, Alberto Cella, Carlo Custodero, Lee Smith, Marina Barbagelata, Stefania Maggi, Mario Barbagallo, Carlo Sabbà, Luigi Ferrucci, Alberto Pilotto

**Affiliations:** 1 Geriatric Unit, Department of Internal Medicine and Geriatrics, University of Palermo, Via del Vespro, 141, 90127 Palermo, Italy; 2 Neuroscience Institute, National Research Council, Padua, Italy; 3 Department of Geriatric Care, Orthogeriatrics and Rehabilitation, E.O. Ospedali Galliera, Genoa, Italy; 4 Department of Interdisciplinary Medicine, Clinica Medica e Geriatria “Cesare Frugoni”, University of Bari “Aldo Moro”, Bari, Italy; 5 Centre for Health, Performance, and Wellbeing, Anglia Ruskin University, Cambridge, UK; 6 National Institute on Aging, National Institute of Health, Bethesda, MD USA

**Keywords:** Multidimensional prognostic index, Frailty, Mortality, Quality of life, ELSA

## Abstract

**Purpose:**

Frailty has been found to be associated with poor quality of life (QoL) in older people, but data available are limited to cross-sectional studies. We therefore aimed to assess the association between multidimensional frailty, determined by Multidimensional Prognostic Index (MPI), with mortality and good QoL expectancy (GQoLE) in a large representative sample of older adults, over 10 years of follow-up.

**Methods:**

In the English Longitudinal Study of Ageing, using the data from 2004–2005 and 2014–2015, MPI was calculated using a weighted score of domains of comprehensive geriatric assessment, i.e., number of difficulties in activities of daily living (ADL) and instrumental ADL, depressive symptoms, number of medical conditions, body mass index, physical activity level, and social aspects. Mortality was assessed using administrative data, GQoLE indicators were used for longitudinal changes in QoL.

**Results:**

6244 Participants (mean age 71.8 years, 44.5% males) were followed up for 10 years. After adjusting for potential confounders, compared to people in the MPI low-risk group, people in the moderate (hazard ratio, HR = 4.27; 95% confidence interval, CI 3.55–5.14) and severe-risk group (HR = 10.3; 95% CI 7.88–13.5) experienced a significantly higher mortality rate. During the follow-up period, people in the moderate and severe-risk groups reported lower GQoLE values than their counterparts, independently from age and gender.

**Conclusions:**

Multidimensional frailty was associated with a higher risk of mortality and significantly lower GQoLE, suggesting that the multifactorial nature of frailty is associated not only with mortality, but also poor QoL.

**Supplementary Information:**

The online version contains supplementary material available at 10.1007/s11136-022-03152-9.

## Plain English summary

Literature regarding frailty and quality of life (QoL) is limited to a few cross-sectional studies. Therefore, using data from the English Longitudinal Study on Ageing (ELSA), we aimed to assess associations between multidimensional frailty, determined by MPI (Multidimensional Prognostic Index), with mortality and good QoL expectancy (GQoLE) indicators, over 10 years of follow-up. Overall, multidimensional frailty was strongly associated with poorer QoL and higher mortality risk. It is thus of importance to screen early for the presence of multidimensional frailty in order to try to improve QoL in older adults.

## Introduction

Frailty is associated with a decline in multiple physiological systems with a resultant elevated vulnerability to stressors [1]. It is widely known that frailty increases the risk of multiple adverse health outcomes in older people, such as disability, falls, hospitalization, institutionalization and, consequently, mortality [2]. Frailty is highly prevalent among older people. For example, a recent study using comprehensive geriatric assessment (CGA) to identify multidimensional frailty found that frailty may affect more than a quarter of older people, with a significant difference across settings [3].

While specific tools for frailty have been developed and studied, there is also evidence that elements of the standard CGA, often collected during geriatric clinical practice, can be used to assess frailty. In this regard, the MPI is commonly used for clinical decision making that captures different aspects of frailty, some of which have been extensively used to study frailty [4, 5]. Although the MPI was originally developed and validated in hospitalized older people [6], it is widely applied in other settings and conditions such as ambulatory clinical settings [7] or community-based studies that have included > 54,000 subjects, to date [8–11].

There is a large body of literature that has consistently found MPI to be associated with several negative outcomes. However, research regarding the impact of higher MPI values, which can be considered a proxy measure of multidimensional frailty, on QoL is still unknown. Previous research has reported that frailty, mainly identified using the physical frailty phenotype, is associated with poor QoL [12]. However, these studies utilized small sample sizes and were of a cross-sectional design, consequently limiting the representativeness of findings [12]. Cross-sectional studies, often failed to include more severe ill patients, introducing a possible “informative censoring” [13]. Moreover, QoL is an important outcome and increasing research has shown that it should be included in epidemiological investigation [14], since one objective of modern geriatric medicine is to implement interventions for increasing living years in good QoL and not only for increasing life expectancy, LE [15].

To date, no study has explored the association between multidimensional frailty and QoL utilizing a longitudinal design. Therefore, the aim of the present study was to investigate associations between multidimensional frailty, assessed by MPI, with mortality and GQoLE indicators, in a large representative sample of older English adults, over 10 years of follow-up.

## Materials and methods

### Study population

This study is based on data from the ELSA between wave 2 (2004–2005) and wave 7 (2014–2015). The ELSA is a prospective and nationally representative cohort of men and women living in England [16]. The ELSA was approved by the London Multicenter Research Ethics Committee (MREC/01/2/91). Informed consent was obtained from all participants. For the aims of this research, we included people older than 60 years, of both genders, since MPI was developed only in older adults [6].

The interviewers made contact with 97% of the households that were issued for ELSA Wave 2 (the household contact rate). The largest component (77%) of non-response was a result of refusals. No specific inclusion and exclusion criteria were applied in the ELSA study [16].

### The ELSA multidimensional prognostic index

The MPI is a common tool for identifying multidimensional frailty in older people. Some common components of MPI were not available in the ELSA study. Therefore, we developed a MPI based on the plasticity of the original MPI measure, termed MPI-ELSA [17].

Originally, the MPI was built according to eight different scales, i.e., disability in basic and instrumental activities of daily living (ADL), using the Katz et al. [18] and Lawton–Brody [19] indexes, respectively, nutritional domain, investigated with the mini-nutritional assessment [20], severity of comorbidities [21], number of drugs taken daily, risk of pressure sores [22], cognitive performance [23], and social aspects [6].

In the ELSA study, the MPI was formulated as follows (Supplementary Table 1):

- Number of difficulties in basic ADL (from 0 to 5) and in instrumental ADL (from 0 to 5), both categorized as 0 (low risk), 1–2–3 (medium risk), 4–5 (high risk);
- Center for Epidemiologic Studies Depression Scale [24] investigating depressive symptoms, categorized as 0 (low risk), 1 (medium risk), ≥ 2 (high risk);
- Number of medical conditions present at baseline, categorized as 0–1–2 (low risk), 3–4 (medium risk), ≥ 5 (high risk);
- Body mass index, categorized as 18.5–24.9 kg/m^2^ (low risk), 25–34.9 (medium risk), < 18.5 or > 35 (high risk);
- Physical activity level [24], categorized as sedentary, low, moderate/high level;
- Social aspects categorized as with family (low risk) and alone (high risk).

This modified MPI, obtained as weighted sum of each domain, ranged from 0.0 (low risk) to 1.0 (highest risk). MPI was categorized into three statistically different risk groups (low risk 0–0.25, moderate risk 0.25–0.43 and severe risk > 0.43), using the RECursive Partition and AMalgamation (RECPAM) algorithm [25], to identify the two best cut-off for creating categories of individuals at different risks of overall mortality.

### Outcomes: mortality and quality of life

Mortality was assessed during the follow-up period using administrative data [16]. QoL was evaluated through the Control, Autonomy, Self-Realization and Pleasure scale (CASP-19) [26]. CASP-19 is a self-completion questionnaire and spans four derived dimensions based on Likert scaled items. CASP-19 has an overall summary measure on a 0–57 scale, with higher scores corresponding to greater well-being [26, 27], A GQoLE indicator was estimated through the Sullivan’s method [28] combining sex- and age-specific LEs obtained from period life tables with sex- and age-specific good QoL prevalence estimated from the ELSA study, with the aim to evaluate the average number of years an individual is expected to live with good QoL.

### Statistical analyses

The data were weighted using the person-level longitudinal weight, core sample, wave 2 (http://www.ifs.org.uk/ELSA). Means and standard deviations (SD) were used to describe quantitative measures, while percentages and counts were used for categorical variables. Characteristics of the study participants at the baseline (wave 2) associated with death during follow-up were evaluated using the *χ*^2^ tests for categorical variables and generalized linear models after testing for homoscedasticity (Levene test) for continuous variables.

The association between MPI risk groups at baseline and mortality during the follow-up observation was investigated estimating survival curves using Kaplan–Meier analyses and the log-rank test. Cox proportional hazard models were used to estimate hazard ratios (HR) and 95% confidence intervals (95% CIs) for MPI and death outcomes, applying a “2-stage” model [17], to adjust for the longitudinal trajectories of MPI during follow-up. First, a mixed model with the outcome MPI, random intercept and slopes including time from the baseline, age, and sex was defined to obtain for each participant estimates for MPI at different time points. These estimates were subsequently considered as exposure in the Cox models with the outcome mortality, adjusting for age, sex, education, marital status, and smoking status. The area under the curves (AUCs) were calculated at different times (3, 6, and 9 years from the baseline), and Harrell’s *C*-index was considered to evaluate the overall performance of the model.

Sullivan method [28], historically used to calculate Disability-Free Life Expectancy was used to construct GQoLE by means of Excel spreadsheets; estimates were calculated by sex and 5-year age classes, and then stratifying also by MPI risk groups. Good QoL was defined using CASP-19 scores higher than the median value observed in the population, considering mortality rates as reported in the WHO abridged life tables for 2005, UK population [29].

All statistical tests were two-tailed, and a *p*-value < 0.05 was considered to be statistically significant. All analyses were performed using the SAS 9.4 and SAS Studio 3.8 software. The.do file is reported in Supplementary Table 3.

## Results

**Fig. 1:**
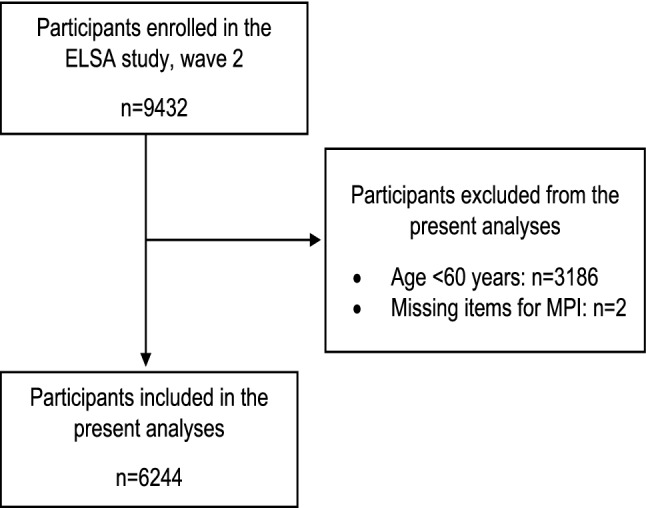
Flow chart of the study (not weighted data)

Of the 9432 participants who took part in wave 2 (baseline) of the ELSA study, 3186 were excluded because they were younger than 60 years, and 2 had missing data in relation to MPI items. Therefore, our analytic study population included 6244 subjects (Fig. 1, not weighted data).

Table 1 shows the data according to survival status during follow-up. The 1392 participants who died during the follow-up period of 10 years had significantly higher MPI values than their counterparts (4852) at the baseline evaluation (mean MPI: 0.39 ± 0.21 vs. 0.25 ± 0.17; *p* < 0.0001). Participants died during the follow-up period were significantly older, more frequently male, less educated, and more frequently current smokers than their counterparts (Table 1).

**Table 1:** Participants’ characteristics according to survival status in the ELSA Study (weighted data)

	Overall population (*n* = 6244)	Alive at follow-up (*n* = 4852)	Dead at follow-up (*n* = 1392)	*p*-value
MPI, mean (SD)	0.25 (0.19)	0.25 (0.17)	0.39 (0.21)	< 0.0001
MPI risk group, *n* (%)				
Low (< 0.25)	3114 (51.4)	2698 (58.4)	417 (29.9)	< 0.0001
Moderate (0.25–0.43)	1845 (30.5)	1335 (28.9)	510 (35.5)	
Severe (> 0.43)	1096 (18.1)	584 (12.7)	513 (35.6)	
Age, years, mean (SD)	71.8 (8.1)	69.9 (7.1)	78.2 (8.0)	< 0.0001
Sex, male, *n* (%)	2696 (44.5)	2000 (43.3)	697 (48.4)	0.0007
Education > 11 years of schooling, *n* (%)	1431 (24.7)	1185 (26.8)	246 (18.0)	< 0.0001
Current smoker, *n* (%)	794 (13.1)	572 (12.4)	222 (15.4)	0.0029
Difficulties in ADL, *n* (%) (*1 missing*)				
0	4517 (74.6)	3672 (79.5)	845 (58.7)	< 0.0001
1, 2, 3	1365 (22.5)	866 (18.8)	499 (34.7)	
4, 5	174 (2.9)	79 (1.7)	95 (6.6)	
Difficulties in IADL, *n* (%) (*1 missing*)				
0	5051 (83.4)	4096 (88.7)	954 (66.3)	< 0.0001
1, 2, 3	888 (14.7)	483 (10.5)	404 (28.1)	
4, 5	117 (1.9)	37 (0.8)	80 (5.6)	
CESD, *n* (%) (*151 missing*)				
0	2201 (37.3)	1827 (40.1)	375 (27.7)	< 0.0001
1	1476 (25.0)	1192 (26.2)	284 (21.0)	
≥ 2	2227 (37.7)	1534 (33.7)	693 (51.3)	
Physical activity level, *n* (%)				
High/moderate	3693 (61.0)	3122 (67.6)	571 (39.7)	< 0.0001
Low	1735 (28.7)	1208 (26.2)	527 (36.6)	
Sedentary	628 (10.3)	287 (6.2)	341 (23.7)	
BMI, *n* (%) (*1252 missing*)				
18.5–24.9 kg/m^2^	1365 (28.4)	1014 (26.4)	351 (36.5)	< 0.0001
25.0–34.9 kg/m^2^	3104 (64.6)	2545 (66.2)	559 (58.2)	
< 18.5 or ≥ 35 kg/m^2^	335 (7.0)	285 (7.4)	51 (5.3)	
Comorbidities, *n* (%)				
0, 1, 2	3403 (56.2)	2808 (60.8)	595 (41.3)	< 0.0001
3, 4	1875 (31.0)	1318 (28.6)	557 (38.7)	
≥ 5	778 (12.8)	490 (10.6)	288 (20.0)	
Cohabitation status, *n* (%) (*1 missing*)				
With family	4131 (68.2)	3338 (72.3)	793 (55.1)	< 0.0001
Alone	1924 (31.8)	1278 (27.7)	647 (44.9)	
CASP score, median (Q1, Q3) (*1952 missing*)	43 (37, 47)	43 (38, 48)	40 (34, 45)	< 0.0001

*SD* standard deviation, *Q1* Quartile 1, *Q3* Quartile 3, *MPI* multidimensional prognostic index, *ADL* activities of daily living, *IADL* instrumental activities of daily living, *CESD* Center for Epidemiologic Studies Depression scale, *BMI* body mass index, *CASP* Control, Autonomy, Self-realization, and Pleasure

Supplementary Fig. 1 graphically shows the association between MPI, in categories, and mortality. Participants at severe risk according higher MPI scores experienced a higher risk of death than the other participants (log-rank *p*-value < 0.0001).

After adjusting for sex, age, education, marital status, and smoking status, compared to people in the low-risk group, people in the moderate (HR = 4.27; 95% CI 3.55–5.14) and severe-risk groups (HR = 10.3; 95% CI 7.88–13.5; *p* < 0.0001) were more likely to die (Table 2). Similar results were evident modeling MPI as a continuous variable.

**Table 2:** Survival analysis for the ELSA Study

	HR	95% CI	*p*-value	Harrell’s *C*-index (95% CI)
MPI, 0.10 increase	1.77	1.68–1.87	< 0.0001	80.8 (79.6–81.9)*
Sex, males vs. females	2.21	1.97–2.48	< 0.0001	
Age, 5 years increase	1.36	1.30–1.43	< 0.0001	
Education > 11 vs. ≤ 11 years of schooling	0.79	0.68–0.91	0.0009	
Marital status, married vs. not married	1.12	0.99–1.27	0.0767	
Present smoker vs. ex- or never	1.83	1.57–2.13	< 0.0001	
MPI, risk group				79.6 (78.4–80.8)**
Moderate vs. Low	4.27	3.55–5.14	< 0.0001	
Severe vs. Low	10.3	7.88–13.5	< 0.0001	
Sex, males vs. females	2.66	2.35–3.01	< 0.0001	
Age, 5 years increase	1.33	1.27–1.40	< 0.0001	
Education > 11 vs. ≤ 11 years of schooling	0.82	0.71–0.95	0.0062	
Marital status, married vs. not married	0.99	0.88–1.12	0.9196	
Present smoker vs. ex- or never	1.80	1.54–2.09	< 0.0001	

*MPI* multidimensional prognostic index, *HR* hazard ratio, *95% CI* 95% confidence interval

*Harrell’s *C*-index for the model with age, sex, education, marital status, smoking status = 76.4 [mean increase on *C* for the model with also MPI of 4.5 (SE 0.004), *p* < 0.0001]

**Harrell’s *C*-index for the model with only age and sex = 75.4 [mean increase on *C* for the model with age, sex, education, marital status, smoking status and MPI of 3.3 (SE 0.004), *p* < 0.0001]

**Fig. 2:**
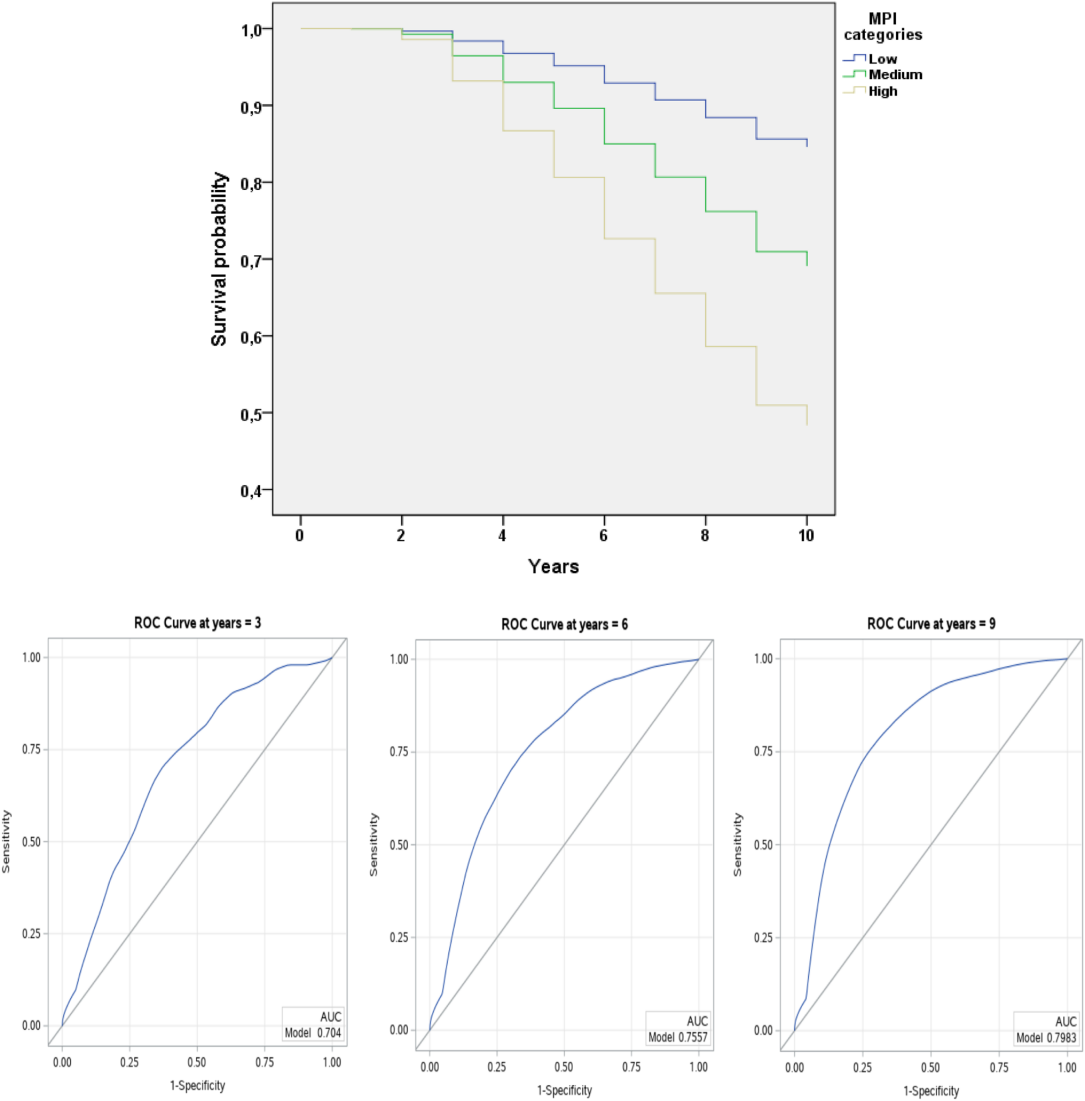
ROC curves at different cut point for the ELSA Study

As shown in Table 2 and in Fig. 2, MPI was overall accurate in predicting mortality. The Harrell’s *C*-index for MPI as increase in 0.10 points was 80.8 (95% CI 79.6–81.9). Similarly, the ROC curves at 3, 6, and 9 years show that the AUCs were 70.4, 75.7, and 79.8, respectively.

**Fig. 3:**
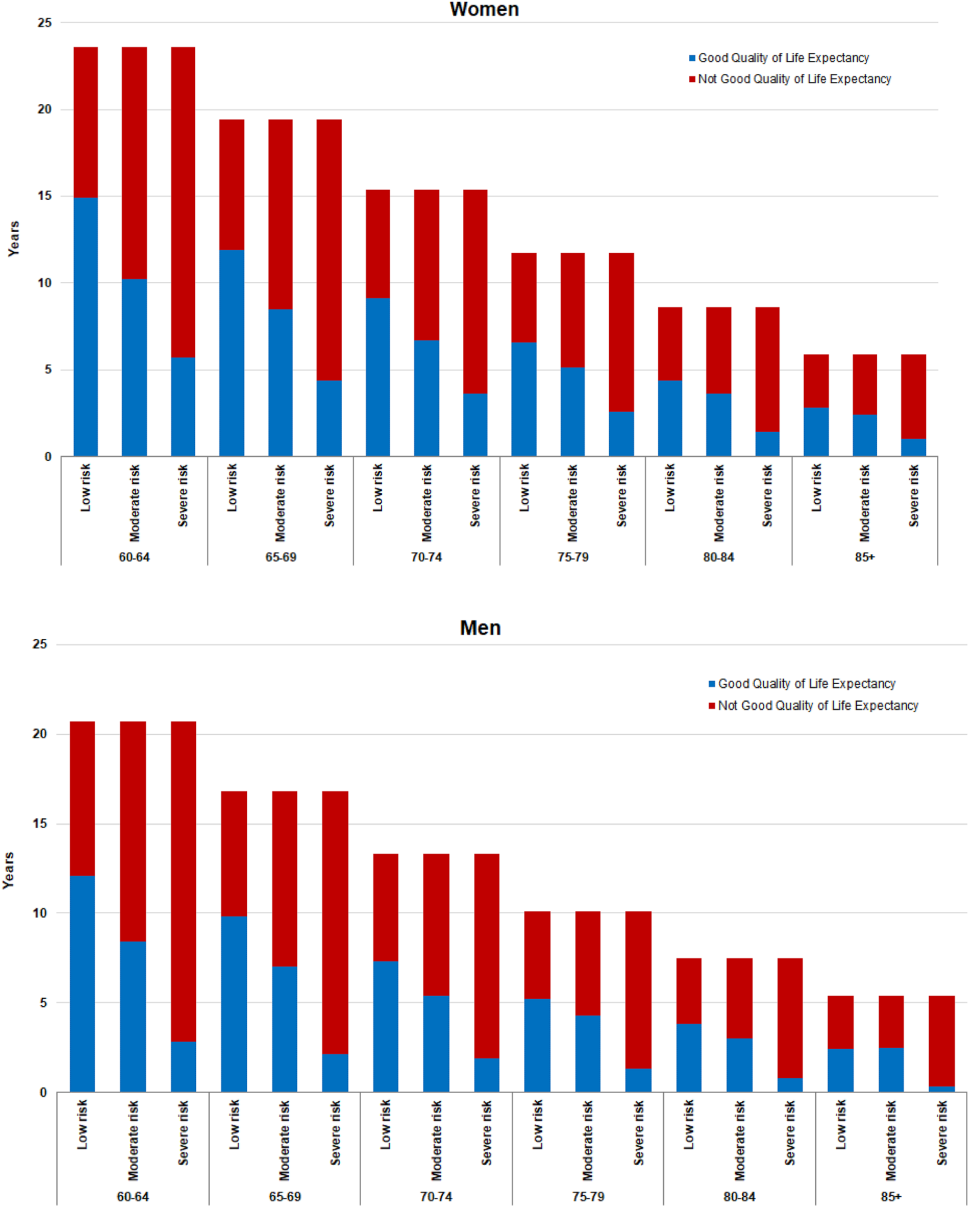
Good Quality of Life Expectancy and Not Good Quality of Life Expectancy (years) by sex, age and MPI risk group for the ELSA study

Finally, as reported in Fig. 3 and in Supplementary Table 2, LE as described by WHO in abridged life tables for 2005 (27) among women aged 60–64 years was 23.6 years, and they can expect to live 11.8 years with good QoL (i.e., approximately 50% of their remaining LE). Moreover, considering MPI groups, women aged 60–64 years in the MPI low-risk group could expect to live 14.9 years with good QoL, those in the moderate group 10.2 and those in the severe-risk group 5.7 years (accounting for 63%, 43% and 24% of their LEs, respectively). Men aged 60–64 years could expect to live a further 20.7 years, of which 11.5 years would be lived with good QoL (50% of their LE). The corresponding GQoLE among men aged 60–64 years in the low MPI risk group was 12.1 years, in the moderate MPI risk group 8.4 and 2.8 years in the severe MPI risk group (accounting for 58%, 41%, and 13% of their remaining life, respectively). Moreover, a trend toward loss of GQoLE was observed with increasing age and more evident in men than in women.

## Discussion

In the present study, including 6244 community-dwelling older subjects living in the UK, we found that higher MPI values at baseline, indicating the presence of multidimensional frailty, were associated with a significant higher risk of mortality over 10 years of follow-up. The accuracy of MPI in predicting mortality was overall good. To the best of our knowledge, this is the first study in a large cohort of older people showing that multidimensional frailty, as assessed by higher MPI values, is associated with worse QoL expectancies during a long follow-up.

Previous studies have reported a significant association between MPI and negative outcomes (in particular mortality) in hospitalized older patients affected by several independent medical conditions [30–35]. However, more recent studies have shown that slightly modified versions of the MPI can predict negative outcomes in community-dwelling older people. For example, in the Swedish National Study on Aging and Care in Kungsholmen (SNACK), higher MPI values at baseline were associated with a higher mortality risk and higher number of days spent in hospital [11]. Similarly, in an Italian population higher MPI values were associated with a higher risk of mortality, with a good accuracy [36]. Finally, in the InCHIANTI study, MPI had a good accuracy in predicting mortality over 15 years of follow-up [17]. Moreover, in other community-dwelling studies, MPI was associated with a higher risk of depression [8], cardiovascular diseases [37], and falls [9] and it is widely known that all these conditions can decrease QoL in older people. Taken together, these findings support the notion that MPI can be used in community-based settings with an accuracy like that found in hospital-based studies and in other settings including long-term care facilities and ambulatory units, since an advantage of the MPI is its plasticity [3, 10].

The current research made another important step forward in better understanding the importance of a CGA-based tool in older people, since higher MPI values at baseline not only predicted higher mortality risk, but also poorer QoL. This finding supports a recent study in approximately 500,000 participants that found considering frailty may change LE estimates by up to 7.2 years [38]. Multidimensional frailty present at the baseline was associated with a dramatical reduction of GQoLE. For example, men and women between 60 and 64 years have approximately 20 years of expected survival, but only half in good QoL. Specifically, participants in MPI 3 (i.e., frail) have approximately 10 years less in good QoL than their counterparts in MPI 1 (i.e., robust) indicating that frailty should be diagnosed early and treated to improving QoL in older people.

It may be suggested that MPI in community-dwelling older people may help in identifying areas which deserve more or less aggressive approaches based on the impaired domains [3]. For example, preventive suggestions (such as vaccinations, healthy diet and increasing physical activity) could be suggested to people in MPI 1 low-risk group in order to avoid the transition to more advanced stages of frailty and consequently to poorer QoL during follow-up. Moreover, in participants already frail, it is important to recommend other interventions, such as deprescribing, resolving social issues or optimizing functional status [3]. The literature suggests that some interventions are effective in improving QoL in frail older people. Multidisciplinary treatment and exercise programs seem to be able to improve QoL in older people affected by frailty, even if the research is still limited to a few studies and with not univocal results [39].

Our findings should be interpreted within the studies limitations. First, the study was of an observational nature. Second, MPI was derived from available data and not according to the original tool: since different approaches for detecting frailty can lead to different findings, other studies are needed to compare the ability of this version of MPI, with the original version. Finally, we did not assess the changes of MPI during the follow-up period.

In conclusion, multidimensional frailty was associated with a significant higher risk of mortality in a large cohort of older English adults, with a good accuracy, confirming that this tool can be used in the community for detecting multidimensional frailty. Moreover, multidimensional frailty was also associated with a relevant reduction in years lived in good QoL, suggesting that the multidimensional approach should be used in older people to allow for the early identification of those that may be at risk of not only a reduced LE but also poor QoL.

## Supplementary Information

Below is the link to the electronic supplementary material.

Supplementary file1 (DOCX 34 kb)

Supplementary file2 (DOCX 32 kb)

## Data Availability

The datasets used and/or analyzed during the current study are available from the corresponding author on reasonable request.
