# Supplementary material for: Multidimensional frailty and quality of life: data from the English Longitudinal Study of Ageing
Source: Qual Life Res. 2022 May 17;31(10):2985–93. doi: 10.1007/s11136-022-03152-9 (PMC9470717; doi:10.1007/s11136-022-03152-9)
Supplement: Supplementary file 1 — Supplementary file1 (DOCX 34 kb) [file 11136_2022_3152_MOESM1_ESM.docx]

**Supplementary Table 1. Calculation of the multidimensional prognostic index in the English Longitudinal Study on Ageing**

| **Domain** | **0 (low risk)** | **0.5 (medium risk)** | **1 (high risk)** |
| --- | --- | --- | --- |
| **Number of difficulties in ADL** | 0 | 1-2-3 | 4-5 |
| **Number of difficulties in IADL** | 0 | 1-2-3 | 4-5 |
| **CESD** | 0 | 1 | >2 |
| **Number of medical conditions** | 0-1-2 | 3-4 | >5 |
| **Body mass index** | 18.5-25 | 25-35 | <18.5; >35 |
| **Physical activity level** | High/moderate | Low | Sedentary |
| **Social** | With family | - | Alone |

**Supplementary Table 2.** Life Expectancy (LE) and Good Quality of Life Expectancy (GQoLE, years and % of the total life expectancy) by sex, age and MPI risk group for the ELSA study

|  | **LE**  **(years)*** | **GQoLE**  **(years and % of LE)** | **GQoLE**  **(years and % of LE)**  **by MPI risk group** | | |
| --- | --- | --- | --- | --- | --- |
|  |  |  | **Low risk** | **Moderate risk** | **Severe risk** |
| **Women** |  |  |  |  |  |
| 60-64 years | 23.6 | 11.8 (50%) | 14.9 (63%) | 10.2 (43%) | 5.7 (24%) |
| 65-69 years | 19.4 | 9.2 (48%) | 11.9 (62%) | 8.5 (44%) | 4.4 (23%) |
| 70-74 years | 15.4 | 6.8 (44%) | 9.1 (59%) | 6.7 (44%) | 3.6 (24%) |
| 75-79 years | 11.7 | 4.8 (41%) | 6.6 (56%) | 5.1 (43%) | 2.6 (23%) |
| 80-84 years | 8.6 | 3.0 (35%) | 4.4 (51%) | 3.6 (42%) | 1.4 (17%) |
| 85 years | 5.9 | 1.9 (32%) | 2.8 (47%) | 2.4 (40%) | 1 (17%) |
|  |  |  |  |  |  |
| **Men** |  |  |  |  |  |
| 60-64 years | 20.7 | 10.4 (50%) | 12.1 (58%) | 8.4 (41%) | 2.8 (13%) |
| 65-69 years | 16.8 | 8.3 (50%) | 9.8 (58%) | 7.0 (42%) | 2.1 (13%) |
| 70-74 years | 13.3 | 6.2 (47%) | 7.3 (55%) | 5.4 (41%) | 1.9 (15%) |
| 75-79 years | 10.1 | 4.4 (44%) | 5.2 (52%) | 4.3 (43%) | 1.3 (12%) |
| 80-84 years | 7.5 | 2.9 (39%) | 3.8 (51%) | 3.0 (51%) | 0.8 (11%) |
| 85 years | 5.4 | 1.9 (35%) | 2.4 (44%) | 2.5 (46%) | 0.3 (5%) |

*: according to abridged life tables for United Kingdom of Great Britain and Northern Ireland, year 2005 (available at https://www.who.int/data/gho/data/indicators/indicator-details/GHO/gho-ghe-life-tables-by-country)

**Figure 1.** Survival curves in the ELSA Study according to MPI categories (weighted data)


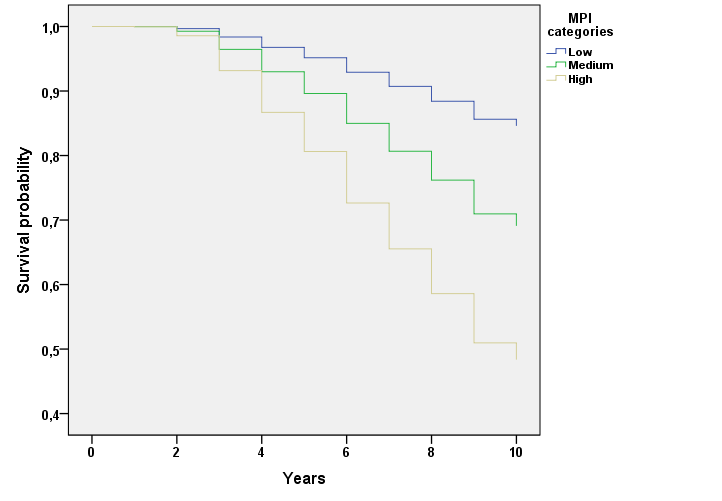


**Supplementary Table 3.** SAS programs

**proc** **freq** data=database;

tables death mpi_categ ragender edu_dic smo_dic adl_mpi iadl_mpi cesd_mpi pa_mpi

bmi_mpi cirs_mpi social_mpi items_mpi

casp2_mio casp_dic

death*(mpi_categ ragender edu_dic smo_dic adl_mpi iadl_mpi cesd_mpi pa_mpi

bmi_mpi cirs_mpi social_mpi items_mpi

casp_dic)/chisq;

weight r2lwtresp;

**run**;

**proc** **means** data=database mean stddev median q1 q3 n;

var mpi r2agey casp2_mio;

class death;

weight r2lwtresp;

**run**;

**proc** **univariate** data=database normal;

var mpi r2agey casp2_mio;

*weight r2lwtresp;

**run**;

**proc** **univariate** data=database;

var casp2;

output pctlpre=P_ pctlpts= **33.3**, **66.6** to **100** by **33.3**;

weight r2lwtresp;

**run**;

**proc** **glm** data= database;

class death;

model mpi r2agey=death;

means death/hovtest=levene welch;

lsmeans death/tdiff stderr;

weight r2lwtresp;

**run**; **quit**;

**proc** **npar1way** data=database wilcoxon;

var casp2_mio;

class death;

**run**;

/*******/

ods output lsmeans=x1;

**proc** **mixed** data=database;

class tte ragender r2agey;

model mpi= tte ragender r2agey

tte*ragender tte*r2agey/outp=p;

random intercept;

weight r2lwtresp;

**run**; **quit**;

ODS OUTPUT CLOSE;

**data** p;

set p;

format pred best4.;

if **0**<=pred <**0.25** then mpi_categ_m=**1**;

else if **0.25**<=pred <=**0.43** then mpi_categ_m=**2**;

else if pred>**0.43** then mpi_categ_m=**3**;

**run**;

**proc** **phreg** data=p;

class ragender (param=ref ref='2')

edu_dic (param=ref ref='0')

mar_dic (param=ref ref='0')

smo_dic (param=ref ref='0');

model tte*death(**0**)= pred ragender r2agey edu_dic mar_dic smo_dic predt ragendert r2ageyt edu_dict mar_dict smo_dict /rl ties=efron;

predt=pred*(tte);

ragendert=ragender*(tte);

r2ageyt=r2agey*(tte);

edu_dict=edu_dic*(tte);

mar_dict=mar_dic*(tte);

smo_dict=smo_dic*(tte);

test_proportionality: test predt;

test_proportionality: test ragendert;

test_proportionality: test r2ageyt;

test_proportionality: test edu_dict;

test_proportionality: test mar_dict;

test_proportionality: test smo_dict;

weight r2lwtresp;

**run**; **quit**;

ods graphics on;

**proc** **phreg** data=p;

class ragender (param=ref ref='2')

edu_dic (param=ref ref='0')

mar_dic (param=ref ref='0')

smo_dic (param=ref ref='0');

model tte*death(**0**)= pred ragender r2agey edu_dic mar_dic smo_dic /rl ties=efron;

hazardratio '0.10 unit change in MPI' pred/units=**0.1000**;

hazardratio '5 unit change in age' r2agey/units=**5**;

weight r2lwtresp;

**run**; **quit**;

ods graphics on; /*SAS STUDIO*/

**proc** **phreg** data=p concordance=harrell(SE)

plots=auc plots(overlay=individual)=roc

rocoptions(at=**3** to **9** by **3**);

model years*death(**0**)=pred ragender r2agey

edu_dic mar_dic smo_dic /rl ties=efron ;

**run**;

/*******/

**proc** **freq** data=p;

tables ragender*age_table*mpi_categ*casp_dic;

weight r2lwtresp;

**run**;

**proc** **freq** data=database;

tables ragender*age_table*casp_dic;

weight r2lwtresp;

**run**;
